# Preparation and epitope identification of duck myxovirus resistance protein monoclonal antibody 4B6 and its expression in duck embryonic tissue

**DOI:** 10.3389/fvets.2026.1840745

**Published:** 2026-08-04

**Authors:** Xinru Song, Shanshan Fan, Zihan Zhu, Chunxue Hao, Yaru Zhang, Hongtao Li, Yongsheng Cao, Bo Ma, Wenlong Zhang

**Affiliations:** 1 College of Veterinary Medicine, Northeast Agricultural University, Harbin, China; 2 College of Basic Medicine, Youjiang Medical University for Nationalities, Baise, China

**Keywords:** duck Mx protein, epitope mapping, innate immunity, monoclonal antibody, prokaryotic expression

## Abstract

**Background:**

Myxovirus resistance (Mx) is a potent antiviral effector induced by interferons, playing a key role in restricting diverse viral infections. However, the specific functions of duck Mx remain poorly understood due to the lack of specific detection reagents.

**Methods:**

In this study, we cloned the Mx gene from ShaoXing duck and expressed and purified its truncated fragment (Mx-a) in prokaryotic cells. Using this purified recombinant Mx-a protein as an immunogen, we generated a monoclonal antibody (4B6) that specifically recognizes native duck Mx.

**Results:**

The antibody, an IgG1 subtype, exhibited high titer and excellent specificity. Further investigation confirmed that mAb 4B6 specifically detects endogenous Mx in duck embryonic tissues by Western blot and recognizes Mx expressed in eukaryotic cells by immunofluorescence. Through fine epitope mapping, the linear epitope recognized by mAb 4B6 was precisely defined as the amino acid sequence “IYFPVPEQ” (residues 67–74), which is highly conserved across different duck species.

**Conclusion:**

The specific monoclonal antibody 4B6 provides an essential tool for further studies on the biological functions, antiviral mechanisms, and innate immune role of duck Mx.

## 1 Introduction

Innate immunity constitutes the first line of defense against infections. Central to this defense are type I (IFN-*α*/*β*) and type III (IFN-*λ*) interferons, which trigger antiviral responses by binding to specific receptors and subsequently inducing the expression of interferon-stimulated genes (ISGs) (1–4). ISG-encoded proteins, such as PKR, 2′,5’-OAS, and the myxovirus resistance (Mx) proteins, establish a cellular antiviral state (5, 6). Among these, Mx proteins are recognized as one of the most potent interferon-induced antiviral effectors, playing a crucial role in restricting diverse viral infections (7–11).

The myxovirus resistance (Mx) gene was first identified in 1962 through its role in conferring innate influenza resistance in A2G mice (12). This was followed by the cloning of human MxA and MxB from interferon-treated cells (13), genes located on chromosome 21, which is homologous to mouse chromosome 16 (14). Orthologs have since been found in nearly all vertebrates and invertebrates—including swine (15), cattle (16–18), avian species (19–21), fish (22–25), abalone (26), and oyster (27)—and are consistently induced by interferon. Mx proteins belong to the dynamin-like GTPase family and share a conserved domain structure: an N-terminal GTPase (G) domain, a middle domain (MD), and a C-terminal GTPase effector domain (GED) (28). Although highly conserved across species, variations in the GTPase domain, subcellular localization, and gene polymorphisms lead to differences in their antiviral activity and mechanism (10, 29). These proteins display broad-spectrum activity against both DNA viruses (30–32) and RNA viruses, typically with greater potency against the latter (33). This broad antiviral activity, particularly against RNA viruses from varied families such as Orthomyxoviridae, Flaviviridae, Bunyaviridae, Paramyxoviridae, has made Mx proteins a focus of extensive study (33).

Compared to other organisms, research on Mx in poultry is limited, with most studies focusing on chicken Mx (2, 34, 35) and only a few on duck Mx. The initial investigation into the duck Mx gene dates back to 1993, when Bazzigher et al. used a probe from a conserved region of the wild duck Mx gene to identify two distinct Mx alleles in NDV- or dsRNA-treated duck embryo cells—the first reported sequence of duck Mx. However, in functional assays, stable expression of duck Mx protein in mouse and chicken cell lines failed to confer resistance against avian-adapted influenza viruses (19). In contrast, more recent studies have demonstrated that duck Mx gene transcription is upregulated upon viral infection (36–40), implicating its potential role in antiviral defense. Although Mx proteins are critical for understanding interferon signaling and viral immune evasion, the precise function of duck Mx remains poorly characterized. This gap largely stems from the lack of specific reagents to detect the duck protein. Consequently, most studies have been limited to analyzing its transcriptional regulation.

This study developed the first monoclonal antibody (4B6) specifically recognizing native duck Mx protein and defined its linear epitope. Using this critical tool, we revealed the tissue-specific expression profile of duck Mx at the protein level following viral infection and identified two putative protein isoforms. These findings provide both a robust research tool and novel insights for future investigation into the precise function and regulatory mechanisms of duck Mx in antiviral innate immunity. Future studies should leverage the 4B6 antibody to delineate the antiviral spectrum, molecular mechanisms, and biological relevance of these duck Mx isoforms.

## 2 Materials and methods

### 2.1 Duck embryos, virus infection, and tissue processing

Twelve-day-old SPF duck embryos (*n* = 3 per group) were inoculated via the allantoic cavity with 10^3^ ELD₅₀ of the duck hepatitis A virus type 3 (DHAV-3) GD strain. At 12 h post-infection (hpi), the heart, liver, stomach, intestine, brain, and muscle were collected, and total RNA was isolated using TRIzol® reagent. For Western blot analysis, tissue samples (200 mg) were homogenized in liquid nitrogen and lysed in RIPA buffer supplemented with 1 mM PMSF. Animal experiments were approved by the Animal Ethics Committee of Northeast Agricultural University.

### 2.2 Cloning of the duck Mx gene

Total RNA (1 μg) was reverse transcribed using Oligo(dT)₁₈ and M-MLV Reverse Transcriptase. The duck Mx gene was amplified by PCR using specific primers (Mx-S/A) designed according to the GenBank sequence NM_001310409 (Table 1). The 2,166-bp product was cloned into pMD-19 T for sequencing.

**Table 1:** PCR primer sequences for Mx and Mx truncated genes.

Primer Name	Sequence (5′-3′)
Mx-S	CGGATATCACCATGGGA ACTACTCAGCATAAC
Mx-A	CCCCTCGAG CTA CAGACAGCTAAAG
Mx-a-S	CGGGATCCATGACTACTCAGCATAAC
Mx-a-A	CCCCTCGAGCTAGTGTTCTGCTGCCTGC
Mx-a-1-S	CCGGAATTCATGACTACTCAGCATAACACAG
Mx-a-1-A	CCGCTCGAGCTATCTTTTGGCCGTTGCTTT
Mx-a-2-S	CCGGAATTCTGCAGTCCTAACCTGATGAA
Mx-a-2-A	CCGCTCGAGCTATTCATCATCCGAGTCTGGTG
Mx-a-3-S	CCGGAATTCATATATTTTCCTGTGCCAGAGC
Mx-a-3-A	CTCGAGCTAGTGTTCTGCTGCCTGC

### 2.3 Construction of expression vectors

A DNA fragment encoding the duck Mx N-terminal region (nucleotides 1—300, designated Mx-a) was amplified by PCR using primers Mx-a-S and Mx-a-A (Table 1), which were designed based on antigenicity predictions from DNASTAR software, with pMD-19 T-Mx as the template. The PCR product was digested and ligated into the BamHI and XhoI sites of the prokaryotic expression vectors pET-30a and pET-32a (Novagen). The recombinant Mx-a protein was expressed in *E. coli* Rosetta (DE3) pLysS by induction with 0.01% IPTG and purified using nickel affinity chromatography. This purified prokaryotically expressed protein was used as the immunogen for monoclonal antibody production. The full-length CDS was cloned into pcDNA3.1(+) for eukaryotic expression. Protein concentration was determined by BCA assay.

### 2.4 Animal immunization and hybridoma generation

BALB/c mice were immunized with 50 μg of purified r30a-duMx-a protein emulsified in Freund’s adjuvant. Booster immunizations were given at two-week intervals. Splenocytes were fused with SP2/0 myeloma cells using PEG. Hybridomas were screened by indirect ELISA using r32a-duMx-a as the coating antigen. Positive clones were subcloned three times by limiting dilution.

### 2.5 Enzyme-linked immunosorbent assay (ELISA)

For screening, 96-well plates were coated with 20 ng/well of purified r32a-duMx protein. After blocking, hybridoma supernatants were added, followed by HRP-conjugated goat anti-mouse IgG. The reaction was initiated with TMB substrate and the absorbance was measured at 450 nm. Antibody isotypes were determined using a mouse monoclonal antibody isotyping kit (ABLAB, China, Cat. No. C06101).

### 2.6 Production and characterization of monoclonal antibodies

Positive hybridoma cells were injected intraperitoneally into pristane-primed BALB/c mice. Ascitic fluid was collected and purified using protein G affinity chromatography. Antibody titer was determined by indirect ELISA with serial two-fold dilutions.

### 2.7 Karyotype and stability analysis

Chromosome number was determined by colchicine treatment followed by Giemsa staining. Antibody secretion stability was assessed by ELISA over 16 consecutive passages.

### 2.8 Western blot analysis

Proteins separated by SDS-PAGE were transferred onto a PVDF membrane using a wet transfer method. The membrane was blocked with 5% (w/v) non-fat dry milk in PBST at room temperature for 2 h, and then incubated overnight at 4 °C with primary antibodies: anti-Mx monoclonal antibody (4B6) diluted 1:100 in PBST; and anti-GAPDH antibody (diluted 1:5000 in PBST; ZSGB-BIO, China, Cat. No. TA-08). After washing, the membrane was incubated with HRP-conjugated goat anti-mouse secondary antibody (diluted 1:5000 in PBST; Bioss, China, Cat. No. bs-0296G) for 1 h at room temperature. Finally, immunoreactive bands were visualized using an enhanced chemiluminescence (ECL) substrate, and the chemiluminescent signals were captured. Images were captured using a GeneGnome (GGNOME-XRQ-NPC, American) imaging system with exposure times optimized for each blot.

### 2.9 Immunofluorescence and epitope blocking assay

To verify that mAb 4B6 recognizes the native duck Mx protein, duck embryo fibroblast (DEF) cells transfected with pcDNA3.1-duMx were fixed with cold acetone for 10 min, permeabilized with 0.1% Triton X-100, and blocked with 3% BSA in PBS. The cells were then incubated with anti-duMx monoclonal antibody (4B6) diluted 1:50 in PBS, followed by incubation with FITC-conjugated goat anti-mouse IgG secondary antibody (ZSGB-BIO, China, Cat No. ZF-0312) diluted 1:5000 in PBS. For epitope mapping, synthetic peptides (at final concentrations of 10 μg/mL and 100 μg/mL in PBS) were pre-incubated with the antibody at 37 °C for 1 h, and the mixture was then applied to pcDNA3.1-duMx-transfected 293 T cells; the subsequent steps were identical to those described above. A reduction or loss of fluorescence signal indicated that the corresponding peptide contained the target epitope. All images were captured using a Zeiss Axio Observer Z1 microscope. Fluorescence intensity was quantified using ImageJ software. Five randomly selected fields were analyzed for each group. The mean fluorescence intensity was measured, and background fluorescence was subtracted. The relative fluorescence intensity was normalized to the mean value of the unblocked control (set as 100%), and the inhibition percentage was calculated. Data are presented as mean ± SD.

### 2.10 Epitope mapping and identification

**Figure 1:**
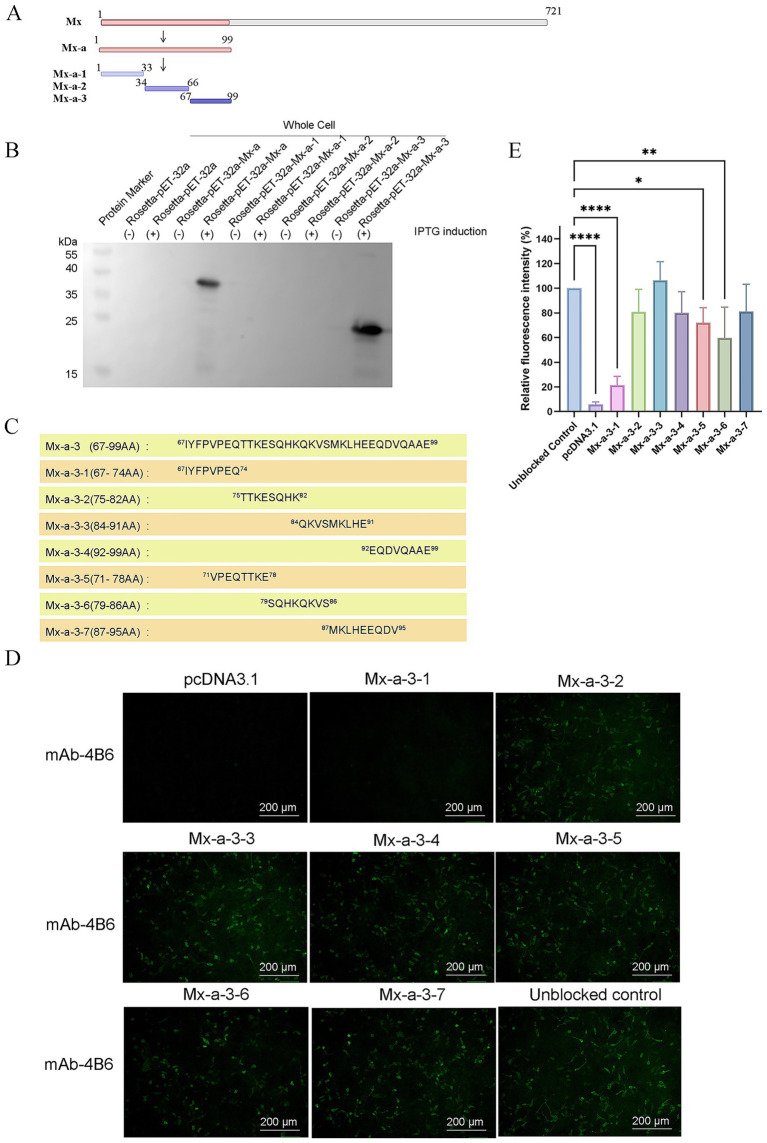
Mapping of the linear epitope recognized by mAb 4B6. **(A)** Schematic representation of the Mx-a protein truncations (Mx-a-1, Mx-a-2, Mx-a-3). **(B)** Western blot analysis assessing the reactivity of mAb 4B6 with the three recombinant Mx-a truncations expressed in *E. coli* Rosetta (DE3) pLysS. The Mx-a fragment served as a positive control. Recognition of full-length duck Mx by mAb 4B6 is shown in Figure 6B. **(C)** Schematic diagram of the seven overlapping synthetic peptides designed to span the Mx-a-3 region identified in panel **(B)**. **(D)** Identification of the minimal epitope by blocking immunofluorescence assay (IFA). 293 T cells overexpressing duck Mx were used. mAb 4B6 was pre-incubated with each synthetic peptide before application. Binding was detected using an FITC-conjugated secondary antibody. **(E)** Quantification of mean fluorescence intensity normalized to the unblocked control (set as 100%). Data are presented as mean ± SD (n=5). The IYFPVPEQ peptide significantly reduced the fluorescence intensity to 18.0 ± 7.4% compared with the control (***P < 0.0001, unpaired t-test).” Please refer to the annotated PDF for details.

The Mx-a fragment was divided by PCR into three overlapping segments (~90 bp each, encoding ~30 amino acids with 8–10 amino acid overlaps) using primers Mx-a-1 S/A, Mx-a-2 S/A, and Mx-a-3 S/A (Table 1). These segments were expressed in pET-32a, and reactive fragments were identified by Western blot. Based on the reactive region identified above, seven overlapping synthetic peptides (8–9 amino acids each, overlapping by 4 residues) (Figure 1C) were chemically synthesized (Sangon Biotech, China) and tested by peptide competition immunofluorescence assay to determine the core epitope.

### 2.11 Statistical analysis

Data are presented as mean ± SD where applicable. No formal statistical testing was performed for qualitative observations.

## 3 Results

### 3.1 Molecular cloning and sequence analysis of the duck Mx gene

A 2,166-bp open reading frame (ORF) was identified in the cloned duck Mx gene, corresponding exactly to the reference sequence (GenBank: NM_001310409). The ORF encodes a 721-amino acid polypeptide with a predicted molecular mass of 81.7 kDa. The N-terminal region contains the conserved tripartite GTP-binding motif and a dynamin-family signature sequence, whereas the C-terminal region harbors a putative leucine-zipper motif common among Mx proteins.

**Figure 2:**
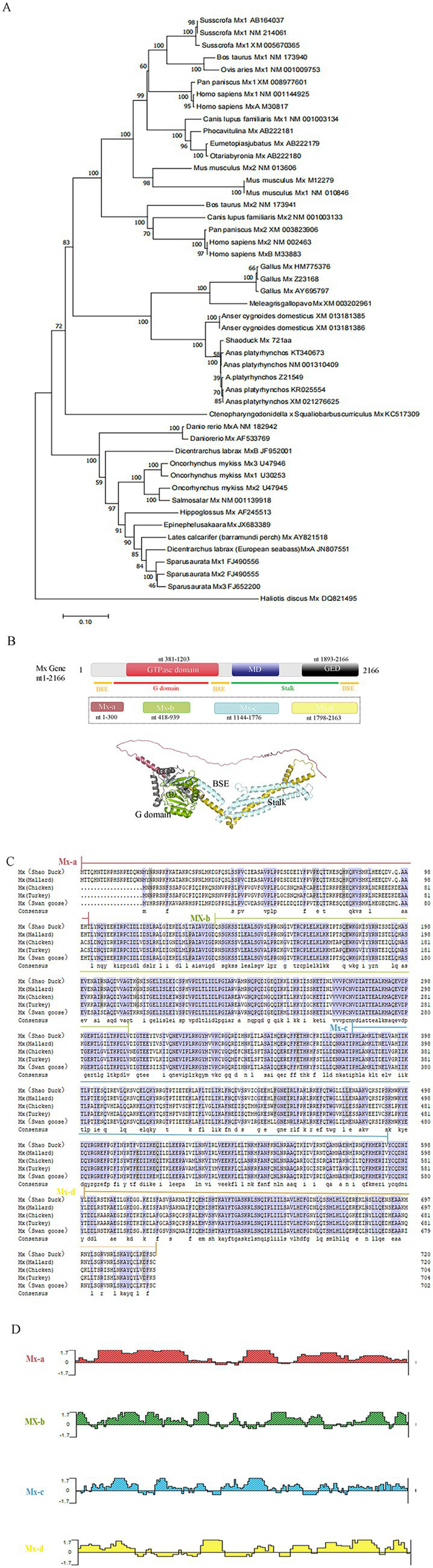
Characterization of truncated Mx gene variants in ShaoXing duck. **(A)** Phylogenetic tree analysis of the Mx gene. **(B)** Schematic diagram of Mx gene truncation in ShaoXing duck. The four truncated gene segments (Mx-a/-b/-c/-d) are illustrated with their primary and tertiary structures, where red represents Mx-a, green Mx-b, blue Mx-c, and yellow Mx-d. **(C)** Amino acid sequence alignment of avian Mx proteins. Mx protein sequences from duck, chicken, turkey, and goose were retrieved from the NCBI database and aligned with ShaoXing duck Mx using DNAMAN software. **(D)** Antigenicity analysis of the Mx-a/-b/-c/-d truncated gene segments in ShaoXing duck.

Nucleotide alignment revealed 99.82% sequence identity between the ShaoXing duck Mx gene and the *Anas platyrhynchos* ortholog, with only four nucleotide substitutions resulting in 99.45% amino acid identity (Supplementary Figure S1). A phylogenetic tree constructed using the neighbor-joining method in MEGA11 (1,000 bootstrap replicates) confirmed that the ShaoXing duck Mx clusters most closely with the *Anas platyrhynchos* Mx gene (Figure 2A).

To design an immunogen for monoclonal antibody production, the full-length Mx gene was truncated into four overlapping segments designated Mx-a, Mx-b, Mx-c, and Mx-d (Figure 2B). Their deduced amino acid sequences were aligned with Mx proteins from *Anas platyrhynchos*, *Gallus gallus*, *Meleagris gallopavo*, and *Anser cygnoides* (Figure 2C). Among these, Mx-a showed the lowest sequence homology to Mx proteins of other species. Antigenicity prediction using DNASTAR indicated that all four truncations possess favorable antigenic potential, with Mx-a exhibiting a significantly higher antigenicity score than the others (Figure 2D). Based on its low cross-species homology and strong predicted antigenicity, Mx-a was selected as the immunogen for subsequent monoclonal antibody development.

### 3.2 Expression and purification of the Mx-a protein

**Figure 3:**
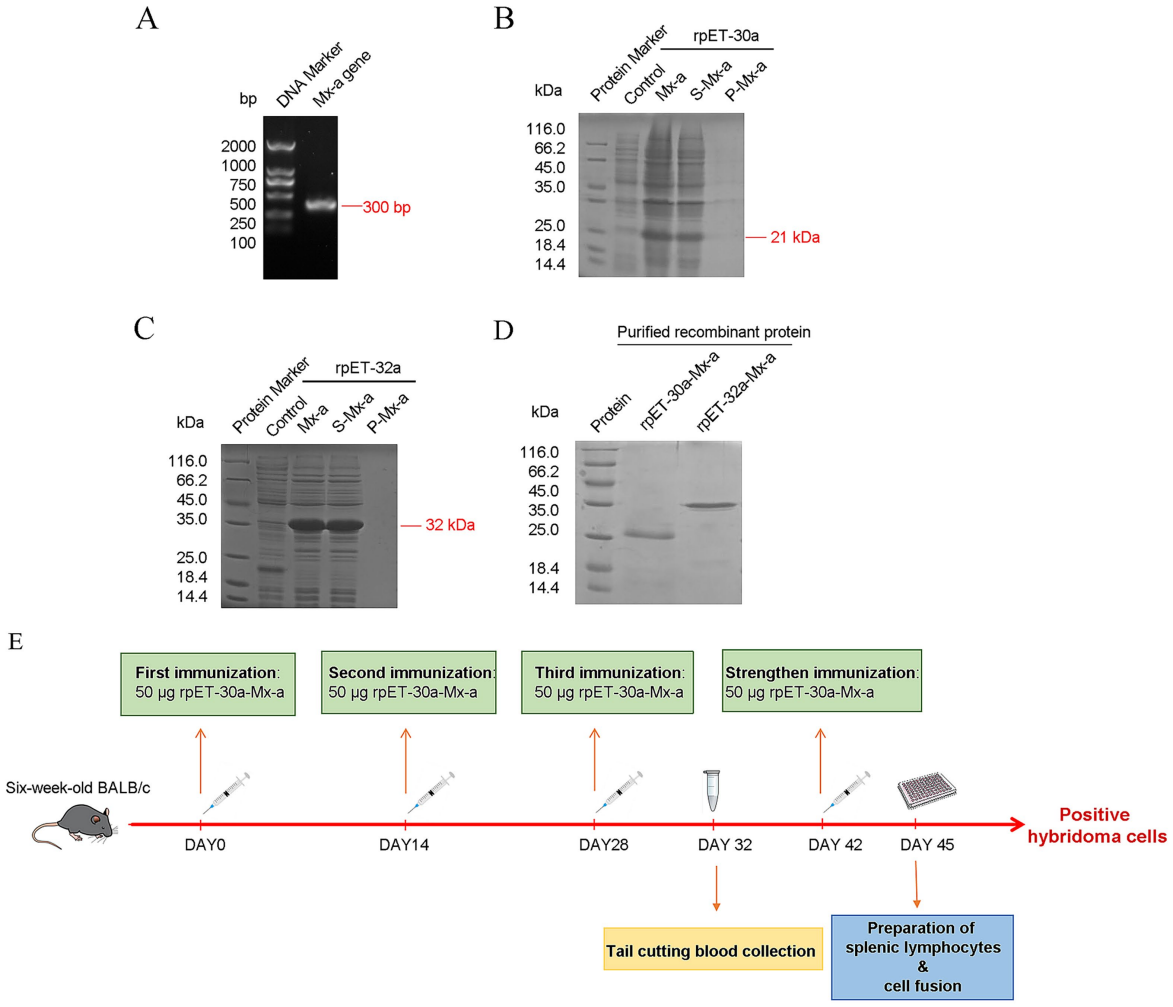
Expression and purification of the recombinant Mx-a protein **(A)** PCR amplification of the Mx-a gene fragment. **(B,C)** SDS-PAGE analysis of Mx-a protein expressed in *E. coli* Rosetta (DE3) pLysS using pET-30a (+) and pET-32a (+) vectors. ‘Control’: cells containing the empty vector. ‘Mx-a’: whole-cell lysate after IPTG induction. ‘S-Mx-a’ and ‘P-Mx-a’: soluble supernatant and pellet fractions after sonication, respectively. **(D)** Purification of the His-tagged Mx-a protein by Ni-NTA affinity chromatography. **(E)** Schematic of the mouse immunization protocol.

The Mx-a gene fragment was amplified by PCR from a plasmid containing the full-length Mx gene (Table 1) to yield a ~ 300 bp product (Figure 3A). Successful cloning of this fragment into the pET-30a (+) and pET-32a (+) vectors was confirmed by restriction digest and sequencing.

Recombinant proteins were expressed in *E. coli* Rosetta (DE3) pLysS upon induction with IPTG. SDS-PAGE analysis of the bacterial lysates revealed distinct bands of approximately 21 kDa and 32 kDa for the pET-30a-Mx-a and pET-32a-Mx-a constructs, respectively, indicating successful expression. Both proteins were produced predominantly in soluble form (Figures 3B,C).

The His-tagged proteins were purified under native conditions using nickel-affinity chromatography. SDS-PAGE of the eluted fractions showed a single prominent band, confirming the acquisition of high-purity recombinant Mx-a protein (Figure 3D). The purified protein from the pET-30a-Mx-a construct (r30a-duMx-a) was used as the immunogen for mouse immunization and subsequent hybridoma generation (Figure 3E).

### 3.3 Screening and stability analysis of hybridoma cells

**Figure 4:**
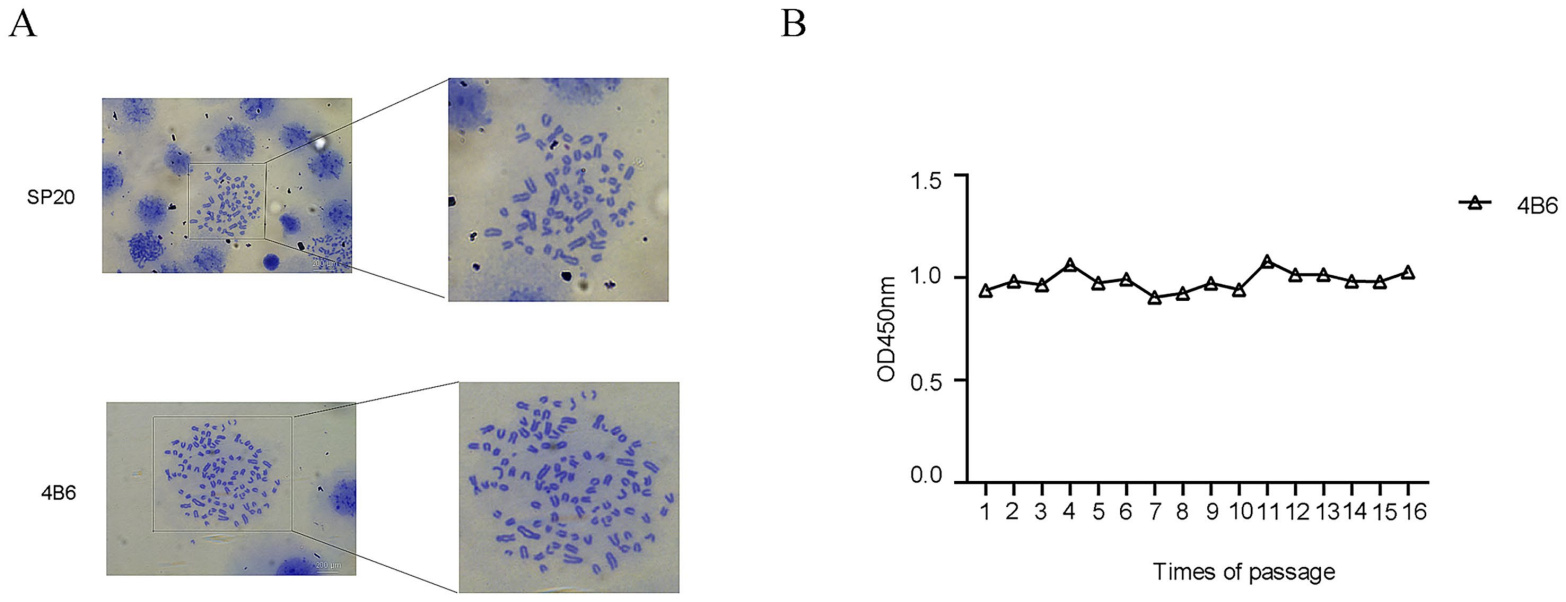
Characterization of hybridoma clone 4B6. **(A)** Chromosome count of the stable hybridoma clone 4B6, determined by the colchicine method. **(B)** Analysis of antibody secretion stability during long-term culture. Clone 4B6 was continuously passaged 16 times. Supernatants from all passages were harvested, and the antibody titer was measured using the established I-ELISA. Positive serum, negative serum, and SP2/0 supernatant served as controls for the assay.

Hybridomas were screened by indirect ELISA using purified r32a-duMx-a as the capture antigen. After three rounds of subcloning by limiting dilution, a stable monoclonal hybridoma, named 4B6, was obtained. Karyotype analysis of clone 4B6 showed a chromosome count consistent with successful fusion (Figure 4A). The antibody production stability of this clone was assessed over serial passages, with ELISA confirming consistent antibody secretion (Figure 4B).

### 3.4 Characterization of monoclonal antibody 4B6

**Figure 5:**
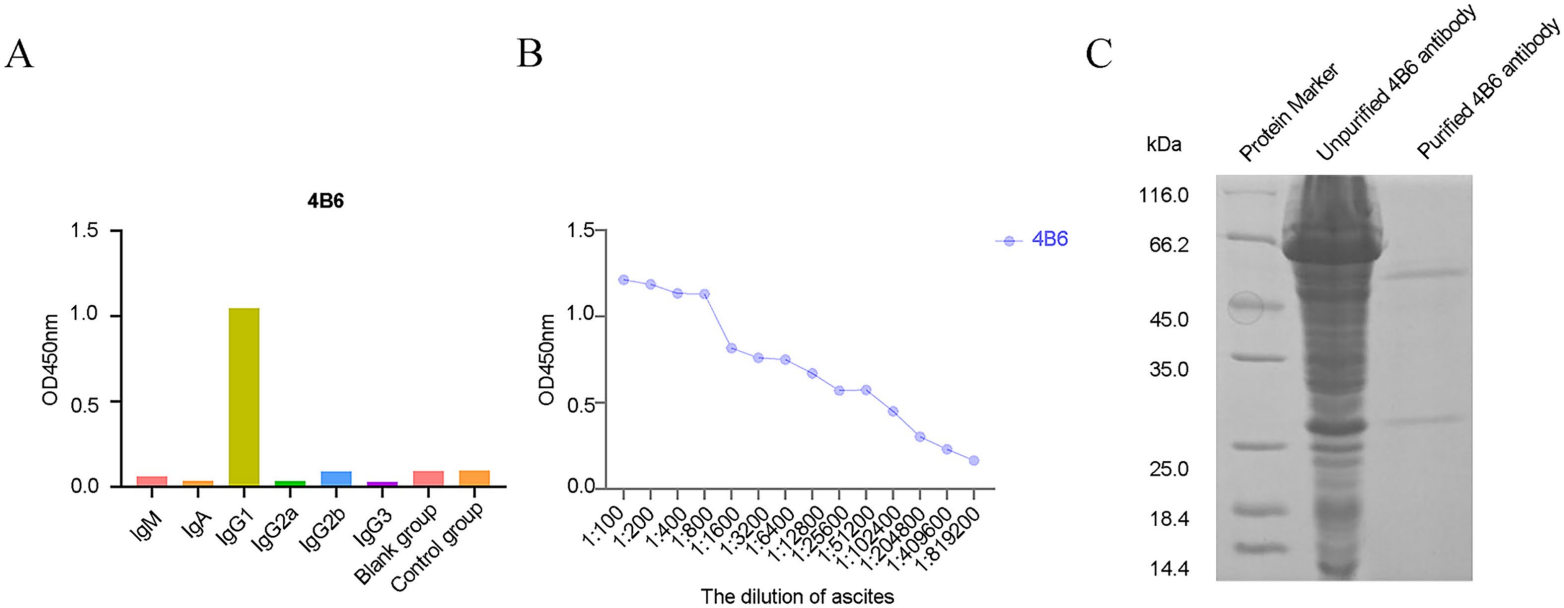
Characterization of monoclonal antibody 4B6. **(A)** Determination of antibody isotype using a commercial mouse monoclonal antibody isotyping kit. **(B)** Measurement of antibody titer in ascitic fluid by indirect ELISA. Ascites was subjected to twofold serial dilutions as the primary antibody. The endpoint titer, defined as the highest dilution yielding a positive-to-negative (P/N) ratio ≥ 2.0 at OD_450_ nm, is shown. **(C)** Assessment of antibody purification. SDS-PAGE analysis under reducing conditions of the effluent from Protein G affinity chromatography confirms the presence of purified antibody heavy and light chains.

The 4B6 monoclonal antibody was identified as an IgG1 subtype using a mouse antibody isotyping kit (Figure 5A). Ascites fluid was produced from mice inoculated with the 4B6 hybridoma, and the antibody titer, as determined by I-ELISA, reached 1:409,600 (Figure 5B). The antibodies were purified from ascites via Protein G affinity chromatography, with SDS-PAGE analysis under reducing conditions confirming the presence of light and heavy chains (Figure 5C). The final concentration of the purified antibody was 1 mg/mL.

### 3.5 Western blot and immunofluorescence analysis of monoclonal antibody specificity

**Figure 6:**
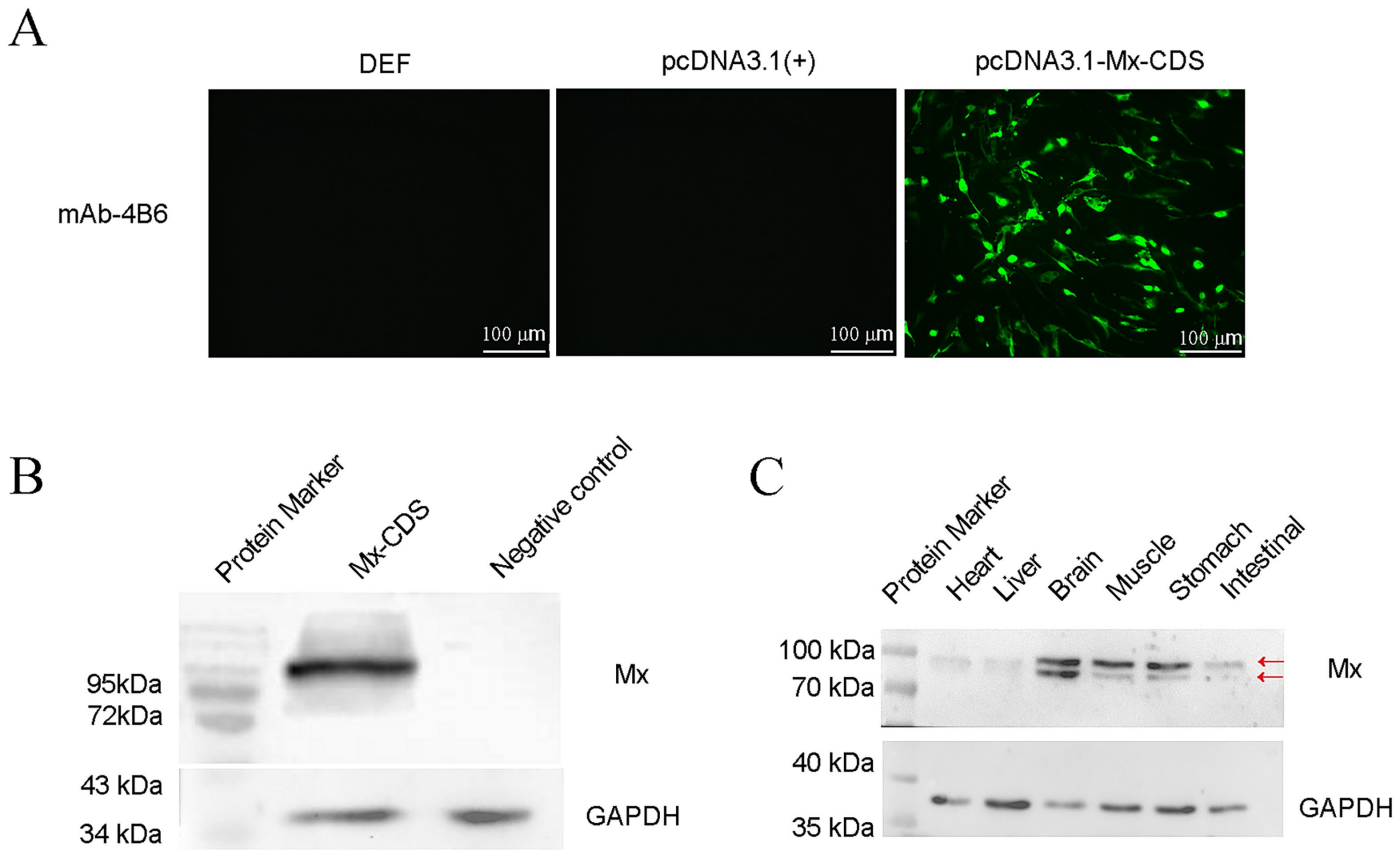
Specificity analysis of monoclonal antibody 4B6. **(A)** Immunofluorescence assay (IFA) of DEF cells transfected with the duck Mx eukaryotic expression plasmid (pcDNA3.1-duMx) or an empty vector control. Cells were incubated with mAb 4B6 (1:50 dilution) followed by an FITC-conjugated goat anti-mouse IgG secondary antibody. **(B)** Western blot analysis of lysates from similarly transfected DEF cells, using mAb 4B6 (1:100 dilution) and an HRP-conjugated secondary antibody. **(C)** Western blot detection of endogenous duck Mx protein in tissues from virus-infected duck embryos. Twenty-day-old SPF duck embryos were inoculated via the allantoic route with 10^3^ ELD₅₀ of DHAV-1 E53. At 12 h post-infection, tissues (heart, liver, brain, muscle, stomach, intestine) were collected and analyzed by western blot using mAb 4B6. Arrows indicate the two distinct bands detected (~82 kDa and ~79 kDa).

The specificity of monoclonal antibody 4B6 was assessed using both immunofluorescence (IFA) and Western blot. IFA of DEF cells transfected with pcDNA3.1-duMx showed bright green fluorescence, and Western blot of the same cells yielded a clear immunoreactive band (Figures 6A,B). These results demonstrate that 4B6 specifically recognizes the ShaoXing duck Mx protein expressed in a eukaryotic system (DEF). Unlike the bacterially expressed truncated fragment used for immunization, the full-length protein produced in eukaryotic cells maintains its native conformation, confirming that mAb 4B6 binds to the properly folded duck Mx protein.

Furthermore, Western blot analysis of tissues from duck embryos harvested 12 h after DHAV-3 GD infection confirmed that 4B6 detects endogenous Mx protein *in vivo*. A protein within the expected molecular weight range (approximately 72–95 kDa) was detected in the heart, liver, brain, muscle, stomach, and intestine (Figure 6C). Mx expression appeared relatively higher in the brain, muscle, and stomach compared with the heart, liver, and intestine. Notably, two distinct bands were consistently detected by 4B6 in tissue lysates. The larger band, with a molecular weight of approximately 82 kDa, was present in all tissues examined. A smaller band of approximately 79 kDa was also observed and was predominantly enriched in tissues showing higher overall Mx expression, namely the brain, muscle, and stomach, suggesting the presence of putative Mx isoforms. Whether this differential expression correlates with viral load in these tissues remains to be determined.

### 3.6 Identification of the 4B6 antigenic epitope

To map the epitope recognized by mAb 4B6, the Mx-a protein was divided into three overlapping subunits (Mx-a-1, Mx-a-2, and Mx-a-3) (Figure 1A), each expressed in *E. coli* Rosetta (DE3) pLysS using the pET-32a vector. Western blot analysis revealed specific reactivity of mAb 4B6 only with the Mx-a-3 subunit (approximately 24 kDa), while the positive control (full-length Mx-a, approximately 32 kDa) was clearly detected. This result confirmed that the epitope recognized by mAb 4B6 is located within the Mx-a-3 region, corresponding to amino acids 67–99. (Figure 1B).

For fine mapping, seven short peptides spanning this region (8–9 amino acids each, overlapping by 4 residues) were synthesized (Figure 1C). A blocking immunofluorescence assay was performed by pre-incubating mAb 4B6 with each peptide before applying it to 293 T cells expressing duck Mx. Fluorescence was completely abolished only by the peptide containing the sequence IYFPVPEQ (residues 67–74), while the other six peptides had no inhibitory effect (Figure 1D). Quantification of mean fluorescence intensity from five randomly selected fields showed an approximately 81.99% reduction in fluorescence upon IYFPVPEQ treatment (Figure 1E). This confirms IYFPVPEQ as the core linear epitope of mAb 4B6.

### 3.7 Analysis of the spatial structure and conservation of antigenic epitopes

**Figure 7:**
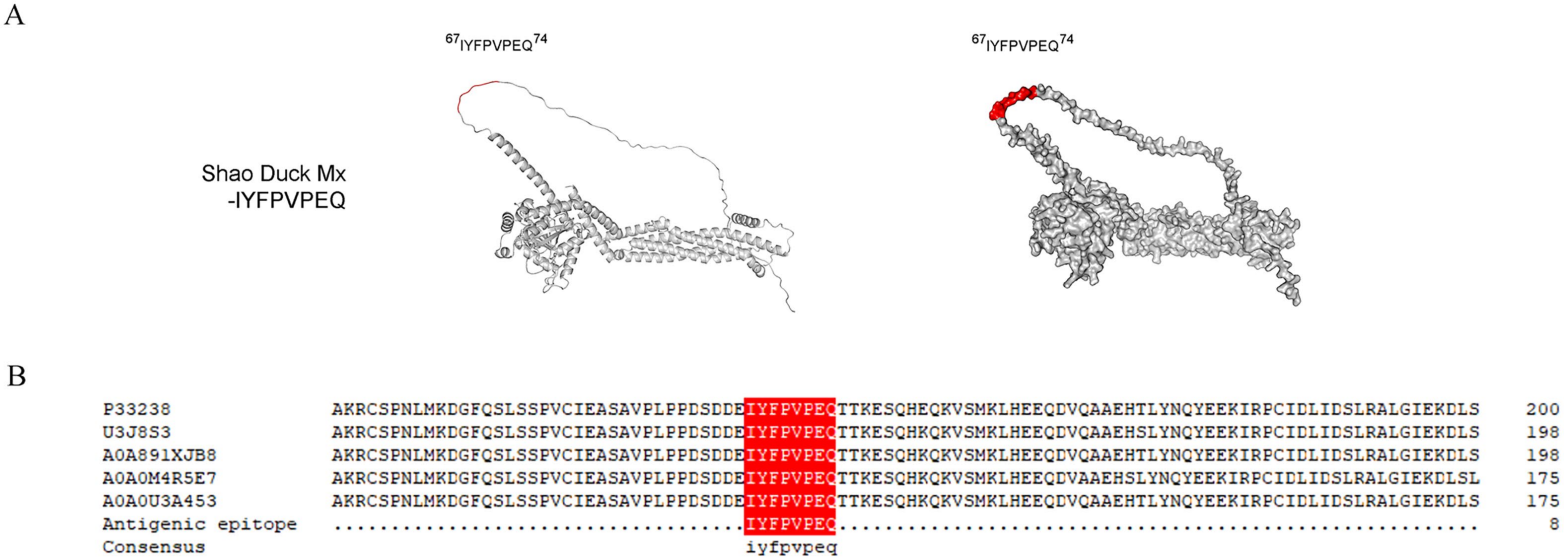
Structural and conservation analysis of the 4B6 epitope **(A)** Spatial visualization of the minimal linear epitope (IYFPVPEQ, aa 67–74) on a structural model of duck Mx protein, rendered in PyMOL and highlighted in red. **(B)** Multiple sequence alignment of the epitope region from Mx proteins of different duck species (UniProt accession numbers provided). The alignment demonstrates the high conservation of the core epitope sequence recognized by mAb 4B6.

The antigenic epitope was visualized using PyMOL software (Figure 7A). Multiple sequence alignment of the identified epitope with Mx proteins from different duck species demonstrated that the epitope sequence recognized by the mAb is highly conserved (Figure 7B). To evaluate the specificity of the identified epitope, a BLASTP search was performed against the *Anas platyrhynchos* reference proteome using the core sequence IYFPVPEQ as the query. The search results confirmed that this sequence is unique to duck Mx protein, with no other duck proteins showing significant matches. This finding further supports the specificity of mAb 4B6 for duck Mx.

## 4 Discussion

Type I interferons (IFNs) exhibit antiviral efficacy by triggering a vast array of downstream interferon-stimulated genes (ISGs) (41). The proteins encoded by these ISGs play a pivotal role in combating and managing pathogens by interfering with various stages of the viral life cycle, thereby inhibiting viral replication or degrading viral components (42). Among these, the Mx protein is particularly notable for its broad antiviral capabilities, serving as a crucial component of the IFN-induced innate immune response in vertebrates and acting as a reliable marker for the biological activity of Type I IFNs. The duck (*Anas platyrhynchos*) is not only a primary natural host for viruses such as Avian Influenza Virus (AIV) but also holds significant economic value as a source of meat, eggs, and feathers (43). In light of this, understanding the characteristics of duck Mx is of paramount importance. However, reports on this subject are scarce, and the specific attributes of duck Mx remain largely unexplored. This study successfully cloned the Mx gene from Chinese ShaoXing ducks for the first time and developed a monoclonal antibody capable of specifically recognizing duck Mx induced by DHAV-3. These findings establish a theoretical and practical foundation for in-depth exploration of the biological characteristics and antiviral activity of duck Mx.

In 1993, Bazzigher et al. successfully cloned and sequenced the 2.3 kb and 2.5 kb polyadenylated mRNAs of the Mx gene from wild duck embryos treated with non-inactivated NDV. The 2.5 kb mRNA variant exhibited two polyadenylation signal sequences, a characteristic attributed to its length exceeding that of the 2.3 kb mRNA. Both the 2.3 kb and 2.5 kb mRNAs contained a large open reading frame, each comprising 2,166 nucleotides and encoding a hypothetical protein of 721 amino acids. Based on nucleotide variations within the open reading frames (ORFs), two distinct groups were identified and designated as *λ* clone 13 (Group I) and λ clone 15 (Group II). There were 11 nucleotide differences between λ clone 13 and λ clone 15, seven of which resulted in amino acid changes (19).

One possible explanation for the apparent discrepancy between the inducible expression of duck Mx upon viral infection and its failure to confer antiviral activity in heterologous mouse or chicken cell lines (19) is that duck Mx may require species-specific co-factors or a permissive intracellular environment—present in duck cells but absent in heterologous systems—to properly target viral components and exert its antiviral function. This hypothesis is consistent with the observation that duck Mx transcription is induced upon viral infection in its native host, supporting its potential role in the duck innate immune response, while also accounting for the lack of activity in cross-species overexpression systems.

In the present study, the Mx gene of ShaoXing ducks was found to share the same ORF as both λ clone 13 and λ clone 15 (Figure 2A). However, it differed from λ clone 15 by 4 nucleotides and 4 amino acids, and from λ clone 13 by 15 nucleotides and 11 amino acids. The ShaoXing duck Mx gene showed higher homology with λ clone 15, with 99.82% identity at the nucleotide level and 99.45% identity at the amino acid level (Supplementary Figure S1). The ShaoXing duck Mx protein presented a typical GTPase structure, featuring GTP-binding motifs fully conserved across all known animal Mx proteins, and a leucine zipper motif identical to those in λ clone 13 and λ clone 15. The presence of two polyadenylation signal sequences in the 3’ UTR of the ShaoXing duck Mx gene—consistent with those reported in λ clone 13 and λ clone 15 (19)—may contribute to the generation of distinct Mx transcript variants. Although the 5’ UTR sequence could not be amplified in this study, its potential variability warrants further investigation, as alternative 5’ UTRs could influence translation initiation and contribute to the Mx putative protein isoforms (~82 kDa and ~79 kDa) detected by mAb 4B6 (Figure 6C).

The monoclonal antibody 4B6 exhibited specific reactivity towards the ShaoXing duck Mx protein expressed by a eukaryotic expression system (Figures 6A,B). Since the immunogen used for antibody generation was a bacterially expressed truncated fragment, the recognition of full-length protein expressed in eukaryotic cells was critical to confirm that mAb 4B6 binds to the native, properly folded duck Mx protein. Western blot analysis confirmed its successful recognition of the native ShaoXing duck Mx protein in the heart, liver, brain, muscle, stomach, and intestine of 20-day-old duck embryos at 12 h post-challenge with DHAV-3 GD (Figure 6C). These findings indicate that 4B6 can serve as an effective reagent for detecting the expression of duck Mx protein in various tissues and organs. This capability opens avenues for exploring the biological characteristics and antiviral activity of the duck Mx protein. Unlike previous studies focusing solely on the transcriptional level of the duck Mx gene (36–40), this study is the first to demonstrate the protein expression pattern in duck embryo tissues. Moreover, Mx protein expression was detected in DHAV-3-infected tissues, suggesting its potential involvement in the host immune response against DHAV-3. This observation is consistent with previous reports showing that DHAV-3 infection activates type I and II interferon responses and upregulates the expression of interferon-stimulated genes (44, 45). However, the absence of direct uninfected controls in this experimental series means that we cannot formally conclude that DHAV-3 induces Mx expression; nevertheless, the detection of Mx in infected tissues is consistent with its role as an ISG and aligns with the published evidence cited above. Notably, the levels of duck Mx protein expressed in the brain, muscle, and stomach were higher than those in the heart, liver, and intestine, highlighting tissue-specific differences in expression levels (Figure 6C). However, as viral load data for these specific tissues were not collected in this study, the relationship between tissue-specific Mx expression and viral replication requires further investigation. 20-day-old duck embryos were selected for two reasons: firstly, SPF duck embryos are more readily available and maintainable than SPF ducks, especially as the embryonic developmental stage minimizes external interference. Secondly, the immune system and tissue development of 20-day-old embryos are sufficiently mature for experimental detection. The dose of 10^3^ ELD_50_ DHAV-3 GD used in this study effectively induced duck Mx protein expression while ensuring embryo survival. This dose is consistent with that commonly used in DHAV-3 infection studies (46).

It is noteworthy that Western blot analysis using mAb 4B6 detected two distinct bands (approximately 82 kDa and 79 kDa) corresponding to duck Mx protein in DHAV-3 GD-stimulated duck embryo tissues (Figure 6C). These bands likely represent putative Mx isoforms, although alternative explanations such as cross-reactivity or proteolytic degradation cannot be formally excluded. This heterogeneity is not an isolated phenomenon; similar patterns were reported in early studies of duck Mx, including in NDV-treated cells and *in vitro* translation products of known cDNA clones (19). Further analysis of these clones reveals three in-frame ATG start codons at nucleotide positions 1, 58, and 115 (Supplementary Figure S1), capable of encoding proteins with calculated molecular weights of approximately 82 kDa, 79.6 kDa, and 77 kDa, respectively. While translation from the first ATG yields the 82 kDa form, it remains to be confirmed whether the smaller isoforms Mx (hereafter referred to as sMx) initiate at the downstream ATGs. This mechanism of generating multiple isoforms from alternative translation initiation sites is precedented, as evidenced by the human MxB protein, which exists in 78 kDa and 76 kDa forms derived from the same transcript, and the two subtypes of human MxB have certain functional differences (47, 48). The short subtype MxB lacks the ability to resist HIV-1 due to the deletion of the N-terminal nuclear localization signal (48), as it starts translation from the second AUG of full-length RNA, which is the 26th amino acid. In the second scenario, duck sMx may have been generated through variable splicing methods such as exon jumping, similar to human MxA resulting in exon 14–16 deletions under variable splicing, and C-terminal frameshift leading to premature termination of translation, resulting in varMxA. The two MxA isomers suggest that the 5′ alternative splicing of Mx may play different roles (49). In recent years, non AUG mediated translation has attracted attention (50). Therefore, there is also a possibility that the start of duck sMx is a non AUG translation start. In summary, the mechanism of duck sMx production and the functional differences between the two isoforms of duck Mx require further investigation.

We acknowledge that the interpretation of these two bands as alternative translation isoforms remains preliminary. The possibility of cross-reactivity of the 4B6 epitope with an unrelated duck protein was excluded by BLASTP analysis (subsection 3.7), but proteolytic degradation or post-translational modification cannot be formally ruled out. Definitive confirmation will require orthogonal approaches such as Mx knockdown or immunoprecipitation followed by mass spectrometry.

Epitope mapping of the monoclonal antibody 4B6 revealed that the antigenic region recognized by 4B6 on the duck Mx protein spans amino acids 67 to 99 (Figure 1B). More precise identification of the epitope was achieved using blocking immunofluorescence assays (IFA) with synthetic peptides at final amounts of 1 μg or 10 μg per 100 μL reaction. The peptide sequence IYFPVPEQ, at both concentrations, completely inhibited specific green fluorescence, indicating successful blocking (Figure 1D). In contrast, the other six tested peptides did not exhibit fluorescence disappearance at either concentration. Typically, epitope identification is performed using ELISA; however, as the synthetic peptides with a molecular weight of approximately 1 kDa were unsuitable for use as coating antigens in ELISA, we opted to the blocking IFA method. This method proved effective. The epitope IYFPVPEQ is located between amino acids 67 and 74.

In summary, this study successfully developed, for the first time, a monoclonal antibody (4B6) that specifically recognizes the natural duck Mx protein and defined its target linear epitope. Utilizing this critical tool, we have unveiled the tissue-specific expression profile of duck Mx at the protein level following viral infection and identified what appear to be two putative Mx isoforms, although orthogonal validation (e.g., knockdown or mass spectrometry) is required to definitively confirm their identity. These findings provide both a robust research tool and novel insights for future investigations into the precise function, regulatory mechanisms, and role of duck Mx in antiviral innate immunity against diverse pathogens. Subsequent work should leverage the 4B6 antibody to delineate the antiviral spectrum, molecular mechanisms, and biological relevance of these duck Mx isoforms.

## 5 Conclusion

This study generated the first monoclonal antibody (mAb 4B6) that specifically recognizes the natural duck Mx protein. The antibody exhibits high specificity and titer, allowing for the first detection of endogenous Mx in duck embryonic tissues. Fine epitope mapping identified the core linear epitope as the conserved sequence “IYFPVPEQ” (aa 67–74). Using this tool, we revealed the tissue-specific expression profile of duck Mx following viral infection and identified two potential protein isoforms, indicative of alternative translation initiation or post-translational modification. These results provide both a critical immunological reagent and novel protein-level insights into the expression and functional diversity of duck Mx. Collectively, this work establishes a foundation for further investigation into the antiviral mechanisms and biological roles of duck Mx in innate immunity. Future studies employing mAb 4B6 should aim to define its antiviral spectrum, characterize the functions of its isoforms, and explore its potential in enhancing disease resistance in poultry.

## Data Availability

All relevant data are included in the manuscript and Supplementary material.
