# Supplementary material for: Preparation and epitope identification of duck myxovirus resistance protein monoclonal antibody 4B6 and its expression in duck embryonic tissue
Source: Front Vet Sci. 2026 Aug 4;13:1840745. doi: 10.3389/fvets.2026.1840745 (PMC13481207; doi:10.3389/fvets.2026.1840745)
Supplement: Supplementary file 1 [file Data_Sheet_1.PDF]

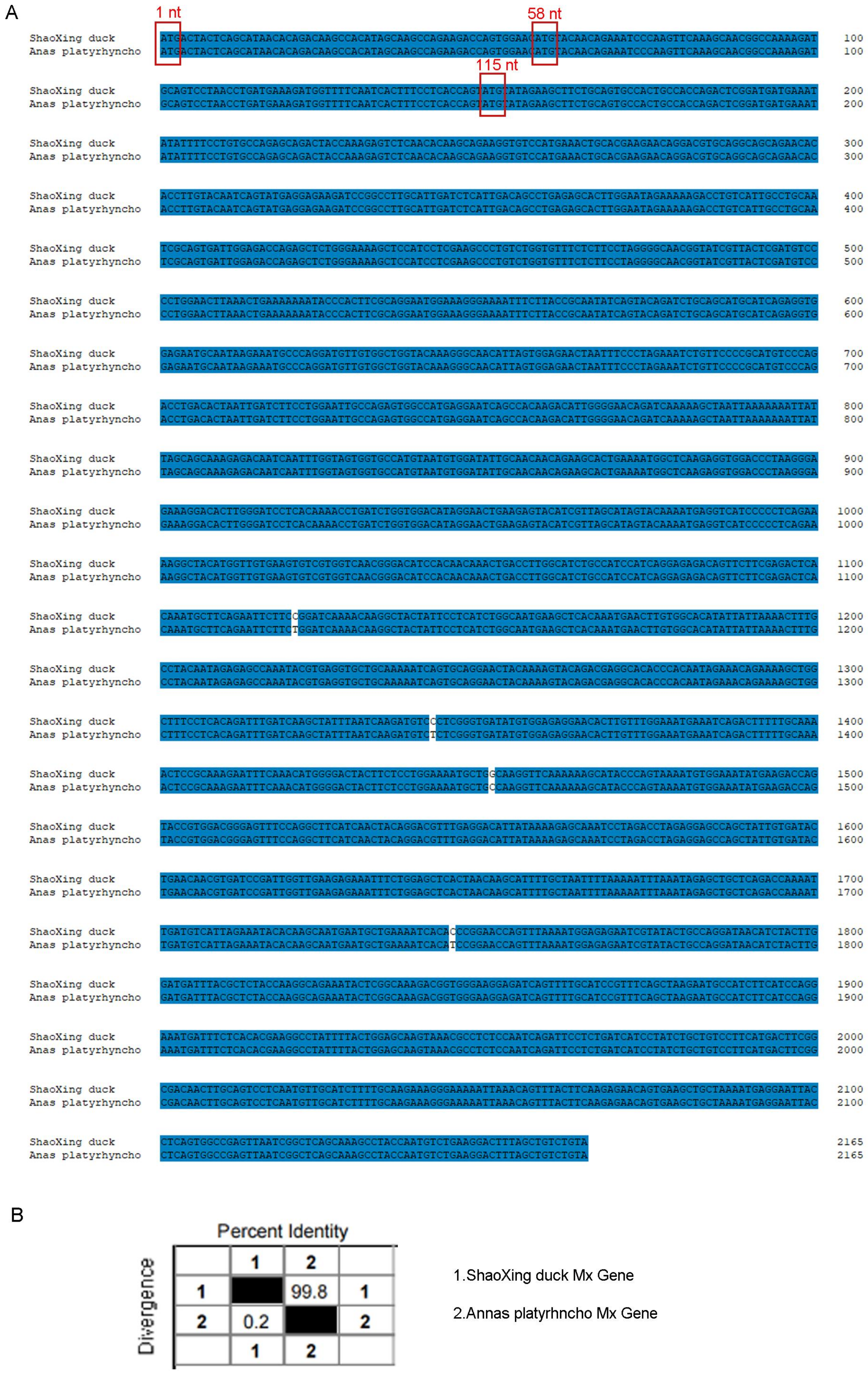
A
1 nt
58 nt
115 nt
ShaoXing duck
Anas platyrhyncho
B
Percent Identity
Divergence
99.8
0.2
1.ShaoXing duck Mx Gene
2.Annas platyrhncho Mx Gene

C

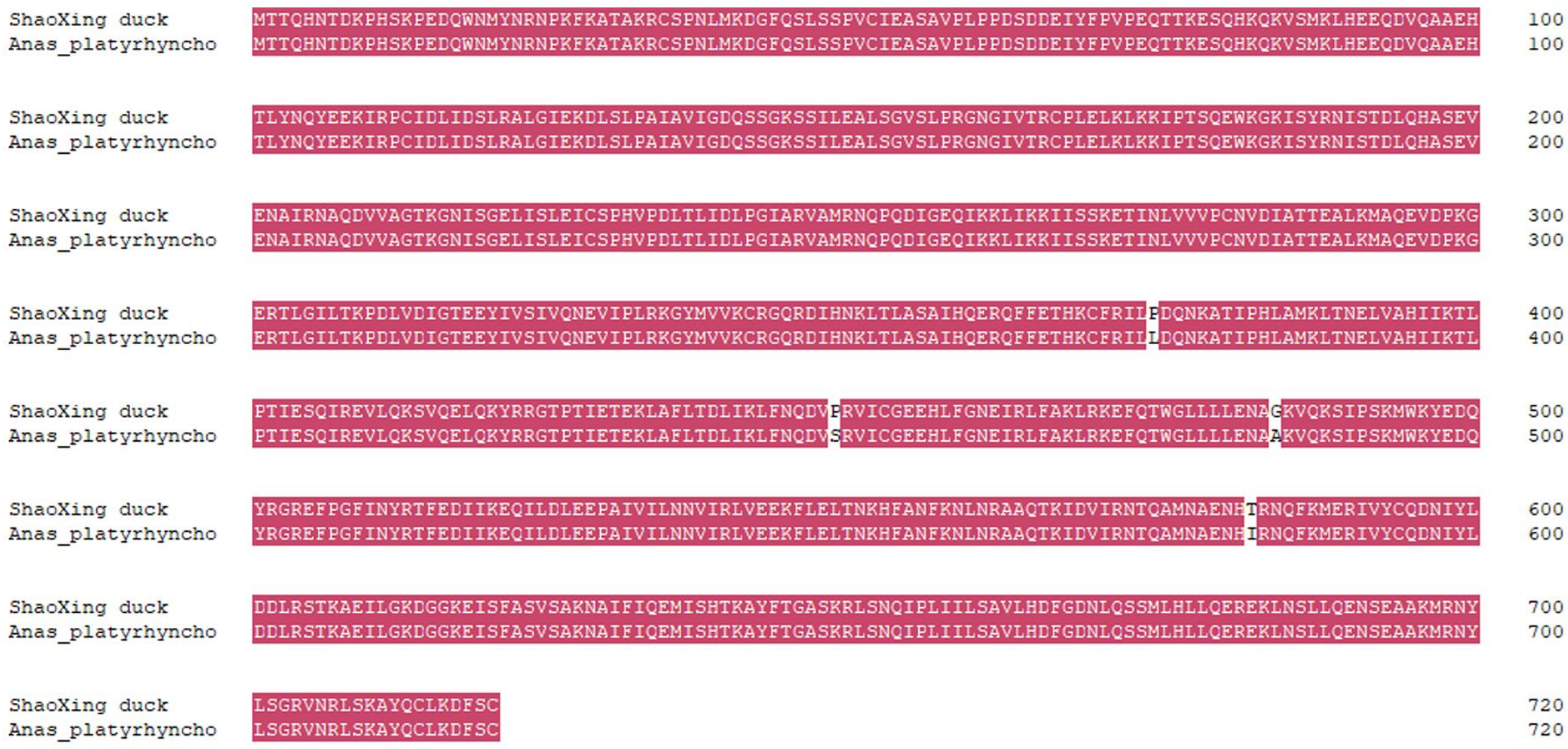

D

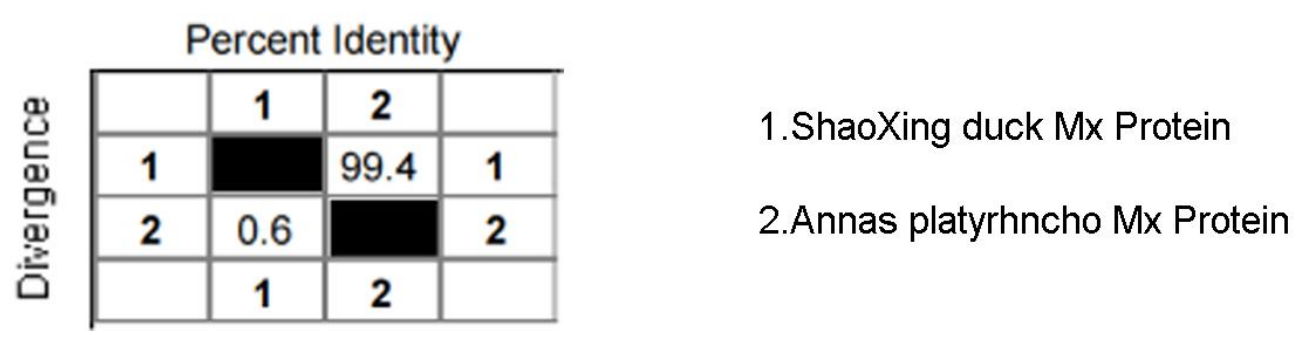

Supplementary Figure S1. Sequence alignment and homology analysis of the ShaoXing duck Mx gene with the *Anas platyrhynchos* ortholog.

(A) Nucleotide sequence alignment. Identical nucleotides are shaded in dark green; gaps are indicated by dashes. The three in-frame ATG start codons (at nucleotide positions 1, 58, and 115) are boxed. (B) Nucleotide homology analysis. (C) Amino acid sequence alignment. Identical amino acids are shaded in dark red; gaps are indicated by dashes. (D) Protein homology analysis. Alignments were performed using DNAMAN software, and homology analyses were performed using Megalign software.
